# Introduce an optimal method of ovarian stimulation in the polycystic ovarian syndrome affected: a randomized controlled trial

**DOI:** 10.1186/s12905-023-02473-2

**Published:** 2023-06-20

**Authors:** Azar Yahyaei, Samira Vesali, Firouzeh Ghaffari

**Affiliations:** 1 Department of Endocrinology and Female Infertility, Reproductive Biomedicine Research Center, Royan Institute for Reproductive Biomedicine, ACECR, Tehran, Iran; 2 Reproductive Epidemiology Research Center, Royan Institute for Reproductive Biomedicine, ACECR, Tehran, Iran

**Keywords:** Polycystic ovarian syndrome, Minimal ovarian stimulation, Mild ovarian stimulation, GnRH-antagonist, Recombinant FSH, Human menopausal gonadotropin

## Abstract

**Background:**

Currently, optimal method of ovarian stimulation (OS) to in-vitro fertilization (IVF) in the patients with polycystic ovarian syndrome (PCOS) is unknown. The present research aims to study the efficiency of minimal-OS method in treatment of infertile patients with PCOS and also the effect of gonadotropin type (recombinant FSH (r-FSH) vs. urinary Human menopausal gonadotropin (u-HMG)) on treatment cycles with GnRH-antagonist.

**Methods:**

In this randomized controlled trial, a total of 120 eligible patients were randomly allocated into four groups of OS to IVF: minimal-OS with r-FSH, minimal-OS with u-HMG, mild-OS with r-FSH and mild-OS with u-HMG. IVF outcomes of groups were analyzed statically.

**Results:**

The statistical analysis showed that there were significant differences among groups regarding stimulation duration (p < 0.0001), number of retrieved oocytes (p < 0.0001), number of obtained embryos (p < 0.0001). There were no statistically significant differences in fertilization rate (p = 0.289) and implantation rate (p = 0.757) among our participants. There were also significant differences among these four groups in terms of clinical pregnancy rate (/ET and /cycles) (p < 0.0001, p = 0.021, respectively) and live birth rate/cycles (p < 0.0001). Also cases of freeze all embryos due to prevention of ovarian hyper stimulation syndrome (OHSS) (p = 0.004).

**Conclusions:**

On the basis of present results the minimal-OS with u-HMG may be one of optimal methods of control OS in the patients with PCOS in respect to serum levels of estradiol on the day of triggering final oocyte maturation, total dose of prescribed gonadotropin, the optimal number of oocytes and embryos obtained, rate of clinical pregnancy and the incidence of OHSS risk.

**Trial registration:**

NCT, NCT03876145. Registered 15/03/2019. Retrospectively registered, http://www.clinicaltrial.gov/ NCT03876145.

## Background

Polycystic ovary syndrome (PCOS) that associated with impaired ovulation is one of the most common causes of infertility with a prevalence rate of 75%. Ovulation induction (OI) is considered one of first options infertility management in women with PCOS history. Due to different causes such as resistance to treatment, failure of OI, assisted reproductive treatment (ART) placed an alternative treatment for these patients. Diversity of ovarian stimulation (OS) protocols for in-vitro-fertilization (IVF), concerns about the risk of ovarian hyper stimulation syndrome (OHSS), higher serum estradiol level, faster endometrial maturation, and such approaches as freeze-all embryos policies are challenges facing IVF in patients with PCOS [1].

Adoption of an optimal OS protocol in these patients to overcome these challenges is highly important. Based on the available scientific evidence, patients with PCOS in the IVF cycle are recommended to undergo OS protocol with gonadotropin-releasing hormone antagonists (GnRH) antagonists. This protocol is preferred over the OS protocol with GnRH-agonist due to reduced OS duration, dose of gonadotropin, and the occurrence of OHSS [2, 3].

In other hand, choosing proper medications can be an important tool to achieve a desired OS outcome and reduce associated complications, such as OHSS. In this regard, the mild ovarian stimulation (mild-OS) and minimal ovarian stimulation (minimal-OS) protocols are cost-effective alternatives [4].

Mild-OS refers to a protocol, which decreases the dose or duration gonadotropin administration in comparison with common protocols in the single OS cycle with GnRH-antagonists [5]. In this definition, mild-OS targets to obtain a maximum of 10 oocytes / time. In the mild-OS protocol, 100–150 IU of gonadotropin is administered at the beginning of the follicular phase. To prevent luteinizing hormone (LH) peak, the GnRH-antagonist is administered in a daily dose after 5–7 days. Some studies have shown that this protocol reduced the gonadotropin dose and OHSS incidence, although it is associated with a considerable increase in the OS cycle cancellation rate because of the poor ovarian response (POR) and the reduced number of obtained oocyte [4]. However, the majority of studies reported a desired rate of implantation after using this protocol [6].

Minimal-OS refers to a protocol, which aims to achieve a maximum of five oocytes. According to the International Society for Mild Approaches in Assisted Reproduction (ISMAAR), minimal-OS aims to obtain 2–7 oocyte. Minimal-OS is performed by administrating anti-estrogenic factors (such as clomiphene citrate (CC)) or aromatase inhibitors (such as letrozole) alone or in combination with a small dose of gonadotropin [4, 7]. The meta-analytical comparison of OS protocols with GnRH-antagonist, with and without CC, has shown a significant difference in terms of live birth rate (LBR), clinical pregnancy rate (CPR), miscarriage, endometrial thickness, and the number of obtaining oocyte. However, a considerable decrease in the incidence rate of OHSS was resulted in the reduction of gonadotropin dose and OS duration which, in turn, caused reduced treatment costs without affecting the clinical outcomes [8].

In addition to the OS and medication protocols, the type of gonadotropin, urinary or recombinant, used for OS influences the IVF cycle outcome. Different types of gonadotropin can be synthesized through purifying human urine such as urinary human menopausal gonadotropin (u-HMG), highly purified HMG (HP-HMG) and recombinant follicle stimulating hormone (r-FSH) [1]. Some studies have reported that these two types of gonadotropin are comparable on the basis of the OS cycle outcome [9]. Some believes that the application of HMG is associated with fewer recovered oocyte and a higher dose of gonadotropin; however, it is similar to r-FSH in terms of CPR [10].

According to evidence, there is an agreement only in using GnRH-antagonists to OS in patients with PCOS; whereas, there is no agreement on the optimal medication and gonadotropin administration for OS to achieve the best fertility outcome in these patients. Randomized control trials (RCTs) on mild/minimal OS protocols in women with PCOS history are scant. The present study aimed to investigate both the effectiveness of minimal-OS, as compared to mild-OS, among PCOS patients and the effect of gonadotropin types in OS cycles with GnRH-antagonist.

## Methods

### Study design

This RCT was carried out on the 116 infertile women who underwent IVF in the Reproductive Biomedicine Research Centre at ROYAN Institute, Tehran, Iran (November2016-May2019). Our study was registered in the international Clinical Trial Website (ClinicalTrials.gov Identifier: NCT03876145). Retrospectively registered at 15/03/2019.

All patients were counseled about the nature of the study and randomization procedure. Also all participants sign consent form and they may drop out of the study at any time. Participating patients were registered in our local ethical committee register that approved the study, and all methods were performed in accordance with the relevant guidelines and regulations.

Due to the lack of a similar study, this pilot study was conducted on four equal-sized groups, each with 30 patients with PCOS. Block randomization method is designed by epidemiologist using STATA software version 13 and the number of blocks considered is 8. The random allocation list for patients is solely available to the epidemiologist. In order to hide the random allocation process, a total of 120 envelopes are prepared, and only the methodologist has been aware of table of random numbers. When the doctor declared the patient’s eligibility, the methodologist provided the doctor with the envelope. The group will be selected and based on the type of group mentioned in the envelope. Each patient participated in the study only once.

Based on the Rotterdam Criteria [11], women with PCOS history who were called for IVF following at least three unsuccessful OI or intrauterine insemination (IUI), infertility duration of at least one year, age 20–38 years, body mass index (BMI) less than 30 kg/m^2^ were included in this study. Exclusion criteria were included, endocrine, autoimmune diseases, hematologic disorders, genetic diseases and chromosomal disorder, history of ovarian and uterine surgery, genital malformations, presence of hydrosalpinx, and uterine fibroids, endometriosis and adenomyosis, history of recurrent miscarriage, and azoospermia-induced infertility.

### Ovarian stimulation

All patients were randomly allocated into four groups: minimal-OS with r-FSH (Minimal-FSH), minimal-OS with HP-HMG (Minimal-HMG), mild-OS with r-FSH (Mild-FSH) and mild-OS with HP-HMG (Mild-HMG). Patients in group of the Minimal-FSH received 100 mg/day CC during the menstrual cycle days, 3rd -7th, and 150 IU/day Gonal-F (Follitropin alfa, Merck Serono, Germany) from the day 7 of the menstrual cycle. Patients in group of Minimal-HMG received 100 mg/day CC during the menstrual cycle days, 3rd -7th and 150 IU/day Merional (Highly Purified Menotropin, IBSA, Switzerland) from the day 7 of the menstrual cycle. Patients in group of Mild-FSH received 150 IU/day Gonal-F from day 3 of the menstrual cycle. Patients in group of Mild-HMG received 150 IU/day Merional from day 3 of the menstrual cycle.

All participants received 0.25 mg Cetrotide (Cetrorelix acetate, Merck Serono, Switzerland) daily since at least one dominant follicle reached a maximum size of 12 mm. Next, 500 µg Ovitrelle (Choriogonadotropin alfa, Merck Serono, Switzerland) was prescribed when at least three follicles reached a size of 17–18 mm. 34–36 h after triggering, ovum pick up was performed. Two to three days after oocyte retrieval, 1–2 fresh embryos at the cleavage stage were transferred into the uterine cavity using the embryo transfer (ET) catheter (Labotect Gmbh, Labor-Technik-Gottingen Kampweg 12, 37,124 Rosdorf, Germany). In all patients, fresh ET was achieved without anesthesia and any difficulty in transfer. Luteal phase was supported by 400 mg twice a day for 14 days of vaginal progesterone (Cyclogest progesterone, Actavis, Barnstaple, EX32 8NS, UK). Progesterone therapy was continued until pregnancy test was performed and in case of positive pregnancy, administration of progesterone suppository continued until 12 weeks of gestation.

Based on the guidelines 2016 from the Royal College of Obstetricians & Gynaecologists (RCOG) [12], triggering final oocyte maturation was done, if there is a risk of OHSS. In this aim, 0.2 mg Decapeptyl (Triptorelin, FERRING, Germany) was administrated and then all embryos were frozen. In all patients, the serum levels of Anti Mullerian Hormone (AMH), FSH, and LH in the 3rd day of menstrual cycle and estradiol and progesterone level on the day of triggering final oocyte maturation were assessed. Two weeks after ET, the serum level of beta-human chorionic gonadotropin (Beta-HCG) was measured. Clinical pregnancy was defined as the presence fetal cardiac activity on vaginal ultrasound four-five weeks after ET. All the pregnant women were followed by the end of pregnancy.

### Outcome measures

The primary outcome was LBR. The secondary outcomes included quality of retrieved oocytes and obtained embryos, fertilization, implantation, clinical pregnancy, miscarriage and OHSS rates. Quality of retrieved oocytes was defined as the total number of Metaphase II (MII) oocytes which reported by the embryologist. Quality of obtained obtaining embryos was assessed based on: (1) the number of cells at Day 2 or Day 3, (2) the amount of fragmentation, (3) the variation in cell size and overall symmetry (perfect, moderately asymmetric, and severely asymmetric in size and shape of the cells), and (4) multinucleation. Fertilization rate was defined as the ratio of 2 pronuclear (2PN) to the total number of inseminated oocytes. Implantation rate was calculated with the number of observed gestational sacs divided by the number of embryos transferred for each patient. Clinical pregnancy was defined as the presence of a gestational sac on ultrasound. Miscarriage was defined as the spontaneous loss of a clinical pregnancy up to 20 weeks of gestation. Live birth referred to the birth of a live fetus after 24 weeks of gestation.

### Statistical analysis

Statistical analysis was performed using SPSS software (version 22; Inc, Chicago, IL, USA). The parameters were compared by use of an analysis of variance (ANOVA) two-way test, followed by Post hoc Tukey simultaneous tests to analyze continuous variables. P_value less than 0.05 was considered statistically significant. The data were analyzed using the two-tailed Student’s t test for independent data, Fisher’s exact test, and two-by-two table between groups where appropriate.

## Results

**Fig. 1:**
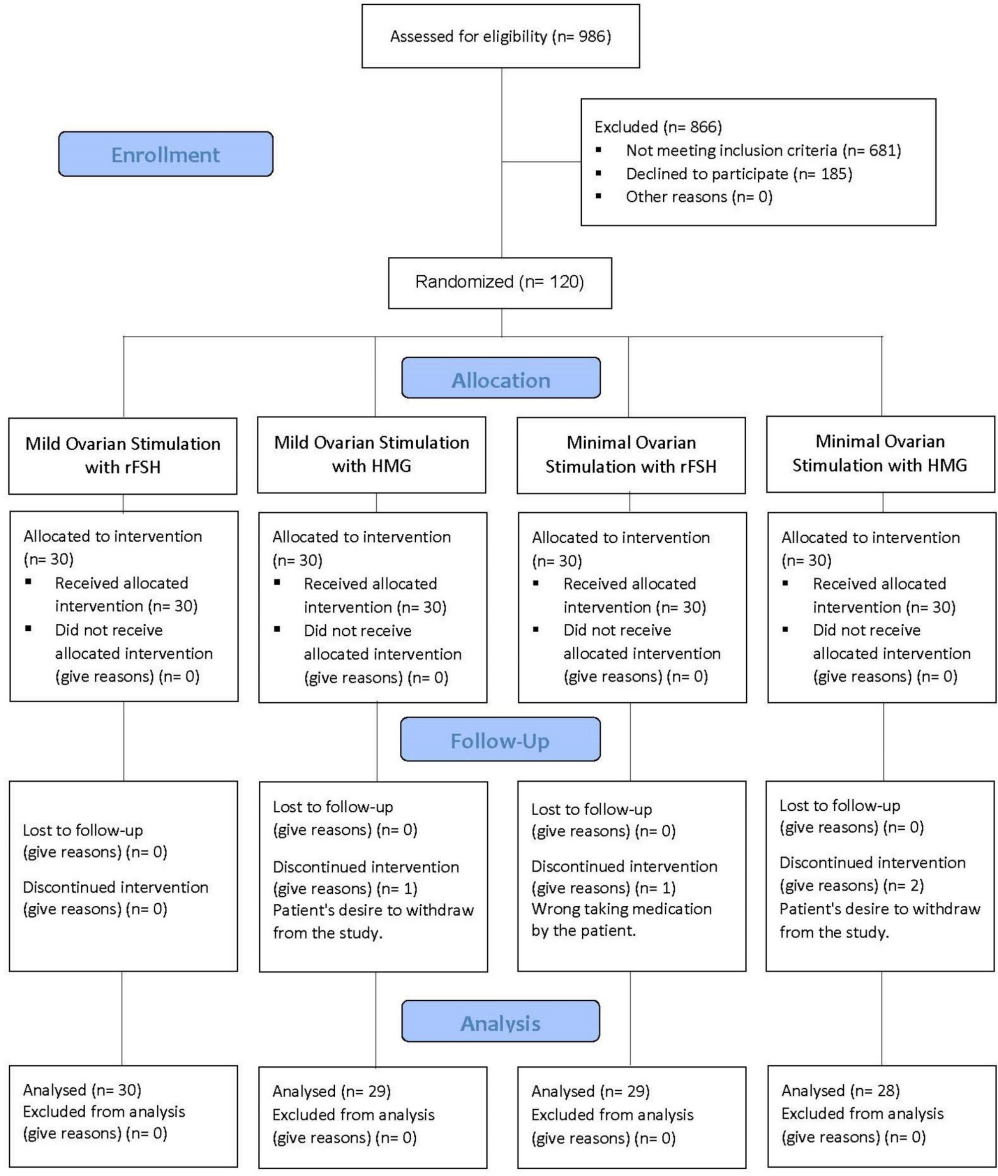
CONSORT Flow Diagram

The flow diagram of the subjects according to the Consolidated Standards of Reporting Trials (CONSORT) guideline is shown in Fig. 1. In total, 120 patients were included in the study. Due to the patient’s desire to leave the study (three cases) and improper use of medication (one case), 116 patients participated. Our groups included, Mild-FSH group: 30 (25.9%), Mild-HMG group: 29 (25%), Minimal-FSH group: 29 (25%), and Minimal-HMG group: 28 (24.1%). No significant differences were observed among these four groups regarding baseline data, demographic data and hormone profile (Table 1).

**Table 1:** Patients’ characteristics in the study groups

	Mild-FSH (n = 30)	Mild-HMG (n = 29)	Minimal-FSH (n = 29)	Minimal-HMG (n = 28)	P_value
**Age (years)**	29.2 ± 3.88	28.21 ± 4.25	28.28 ± 4.52	29.14 ± 4.79	0.723
**BMI (kg/m** ^**2**^ **)**	26.77 ± 3.01	26.12 ± 3.17	26.17 ± 3.41	25.82 ± 3.38	0.726
**Duration of infertility (years)**	6.05 ± 3.07	5.38 ± 2.68	4.86 ± 2.77	5.34 ± 2.46	0.431
**Causes of infertility**					0/806
**PCOS**	18 (60)	18 (62.1)	17 (58.6)	14 (50)	
**PCOS + Male factor**	12 (40)	11 (37.9)	12 (41.4)	14 (50)	
**Type of infertility**					0.734
**Primary**	28 (93.3)	27 (93.1)	26 (89.7)	24 (85.7)	
**Secondary**	2 (6.7)	2 (6.9)	3 (10.3)	4 (14.3)	
**Menstruation**					0.96
**Regular**	11 (36.7)	14 (48.3)	5 (17.2)	10 (35.7)	
**Irregular**	19 (63.3)	15 (51.7)	24 (82.8)	18 (64.3)	
**PCOS Phenotype** ^*^ **A** **C** **D**					0.129
	12 (40)	12 (41.4)	18 (62.1)	7 (25)	
	14 (46.7)	11 (37.9)	9 (31)	17 (60.7)	
	4 (13.3)	6 (20.7)	2 (6.9)	4 (14.3)	
**Hormonal profile**					
**FSH** **(IU/L)**	5.19 ± 0.39	5.31 ± 0.26	5.83 ± 0.31	5.21 ± 0.35	0.489
**LH** **(IU/L)**	6.55 ± 0.61	6.61 ± 0.69	7.35 ± 0.63	6.93 ± 0.66	0.375
**Prolactin** **(ng/mL)**	117.91 ± 22.14	117.59 ± 32.02	116.46 ± 36.99	117.95 ± 29.37	0.654
**TSH** **(IU/mL)**	1.76 ± 0.11	1.58 ± 0.14	1.68 ± 0.17	1.47 ± 0.56	0.172
**AMH** **(ng/mL)**	8.19 ± 0.86	9.31 ± 0.77	10.97 ± 0.97	10.35 ± 0.98	0.136

Values are reported as means ± standard deviations or numbers (percentages)

* Phenotype A: PCO morphology, Oligo-anovulatory, Hyperandrogenism.

Phenotype C: PCO morphology, Oligo-anovulatory.

Phenotype D: PCO morphology, Hyperandrogenism

The statistically higher dose of gonadotropin administered was observed in the Mild-HMG and Mild-FSH groups in comparison with groups of Minimal-FSH and Minimal-HMG (p < 0.0001); there is a significant difference between Mild-HMG group and Mild-FSH group (p < 0.0001), and between Mild-HMG group and Minimal-HMG group regarding prescribed dose of gonadotropin (p < 0.0001) (ANOVA followed by Tukey’s post hoc test). There was a significant difference among the four groups regarding duration of OS (day) (p < 0.0001). We observed the lowest stimulation duration in the Mild-FSH group (p < 0.0001) (ANOVA followed by Tukey’s pos thoc test). Significantly higher estradiol level on the day of triggering final oocyte maturation was detected in the Minimal-FSH group, while Mild-HMG group and Minimal-FSH group showed different status, (p = 0.01) (p = 0.019) respectively (ANOVA followed by Tukey’s post hoc test). Significantly higher progesterone level on the day of triggering final oocyte maturation was in favor of Minimal-FSH group (p = 0.007) (Table 2).

**Table 2:** Ovarian stimulation cycle information in the study groups

	Mild-FSH (n = 30)	Mild-HMG (n = 29)	Minimal-FSH (n = 29)	Minimal-HMG (n = 28)	P_value
**Prescribed dose of gonadotropin (IU)**	1477.5 ± 283.5	1900.5 ± 579.75	1207.5 ± 266.25	1315.5 ± 381	< 0.0001
**Number of** **GnRH-antagonist (ampoule)**	4.63 ± 1.29	5.07 ± 1.82	4.28 ± 0.96	4.39 ± 1.37	0.151
**Duration of ovarian stimulation (day)**	10.67 ± 1.49	12.14 ± 2.27	12.76 ± 1.33	12.93 ± 1.78	< 0.0001
**Endometrium thickness** ^*^ **(mm)**	9.26 ± 1.55	10.27 ± 1.38	9.77 ± 1.62	9.63 ± 1.73	0.119
**Estradiol** ^*^ **(pg/ml)**	3663 ± 458.35	3100.48 ± 282.12	4744.38 ± 312.31	3145.32 ± 458.43	0.007
**Progesterone** ^*^ **(ng/ml)**	1.24 ± 0.24	1.02 ± 0.23	2.33 ± 0.42	1.11 ± 0.20	0.007

Values are reported as means ± standard deviations

* On the day of triggering final OOCYTE maturation

As depicted in Table 3, the mean number of oocytes retrieved per patient was statistically higher in the Minimal-FSH group in comparison with Mild-HMG group (p < 0.0001) and Minimal-HMG group (p < 0.0001). Post hoc Tukey simultaneous tests indicated that the mean number of MII oocytes was statistically significantly higher in the Minimal-FSH group in comparison with Mild-HMG group (p < 0.0001) and Minimal-HMG (p < 0.0001). There was a significant difference among these four groups in the mean number of 2PN (p = 0.006). Also the Minimal-FSH group had statistically significantly higher mean number of 2PN in comparison with Mild-HMG group (p < 0.038) and Minimal-HMG group (p < 0.011).

**Table 3:** Ovarian stimulation cycle Outcomes in the study groups

	Mild-FSH (n = 30)	Mild-HMG (n = 29)	Minimal-FSH (n = 29)	Minimal-HMG (n = 29)	P_value
**Number of OOCYTEs Retrieved**	21.2 ± 12.3	14.23 ± 6.94	26.28 ± 12.83	11.48 ± 9.04	< 0.0001
**Metaphase II**	18.27 ± 12.17	11.88 ± 6.30	23.76 ± 12.54	10.69 ± 9.37	< 0.0001
**Metaphase I**	0.93 ± 0.34	0.69 ± 0.22	0.63 ± 0.23	0.42 ± 0.13	0.505
**Germinal Vesicle (GV)**	0.73 ± 0.2	0.88 ± 0.24	1.31 ± 0.3	0.69 ± 0.25	0.312
**Degeneration**	0.53 ± 0.18	0.42 ± 0.16	0.32 ± 0.14	0.12 ± 0.07	0.236
**Number of 2PN**	11.6 ± 8.19	8.27 ± 5.8	13.52 ± 7.74	7.38 ± 6.41	0.006
**Number of Embryos Obtained**	13.09 ± 8.61	8.38 ± 5.35	14.41 ± 7.94	7.04 ± 6.53	< 0.0001
**Excellent**	4.03 ± 0.73	1.54 ± 0.32	5.59 ± 0.85	3.31 ± 0.93	0.003
**Good**	5.33 ± 0.84	3.73 ± 0.65	5.10 ± 0.76	2.73 ± 0.54	0.042
**Fair**	2.73 ± 0.65	2.58 ± 0.62	3.21 ± 0.69	1.00 ± 0.30	0.066
**Poor**	0.93 ± 0.31	0.5 ± 0.22	1.03 ± 0.32	0.77 ± 0.36	0.638
**Number of Embryos Transfer**	1.85 ± 0.15	1.70 ± 0.17	2 ± 0	1.90 ± 0.09	0.802
**Number of Frozen Embryos**	10 ± 1.38	6.04 ± 0.86	11.31 ± 1.11	5.85 ± 1.23	< 0.001
**Fertilization rate**	0.64 ± 0.04	0.68 ± 0.05	0.59 ± 0.04	0.69 ± 0.05	0.289
**Implantation rate**	0.88 ± 0.24	0.83 ± 0.17	0.5 ± 0	0.7 ± 0.12	0.757
**Cycle cancelation rate** ^**1**^	0	3 (10.34)	0	3 (10.71)	0.827
**Freeze all embryos** ^**2**^	23 (76.66)	17 (58.62)	27 (93.10)	15 (53.57)	0.004
**Clinical pregnancy rate/ ET (%)**	57.14	33.33	50	50	< 0.0001
**Clinical pregnancy rate/ Cycles (%)**	13	10	3	17	0.021
**Miscarriage rate/ ET (%)**	28.57	0	0	20	0.199
**Miscarriage rate/ Cycles (%)**	6.66	0	0	7.14	0.779
**Miscarriage rate/ pregnancy (%)**	50	0	0	40	0.199
**Live birth rate/ ET (%)**	28.57	33.33	50	30	0.896
**Live birth rate/ Cycles (%)**	6.66	10.34	3.44	10.71	< 0.0001

Values are reported as means ± standard deviations or numbers (percentages)

^1^ Due to inadequate ovarian response

^2^ Due to prevention of OHSS

There was a significant difference among these four groups for the mean number of embryos (p < 0.0001). The Minimal-FSH group showed statistically significant higher mean number of embryos compared to Mild-HMG group (p < 0.015) and Minimal-HMG group (p < 0.002); also the mean number of frozen embryos was similar. Frequency comparison of cases of freeze all embryos due to prevention of OHSS among four groups was significantly different (p = 0.004). There were also significant differences among these four groups in terms of clinical pregnancy rate (/embryo transfer and /cycles) (p < 0.0001/ p = 0.021, respectively) and live birth rate /cycles (p < 0.0001).

## Discussion

The present RCT was done to reach the optimal OS protocol for IVF in patients with PCOS, and based on the findings of the present study, the minimal-OS with u-HMG is one of optimal methods of control OS in this patients.

The effectiveness of OS in the ART cycles has been investigated in several studies by considering the pivotal role of OS protocol (GnRH-antagonist vs. agonist), medication protocol (mild-OS vs. minimal-OS), and the type of gonadotropin administered (r-FSH vs. HMG) [6, 8–10, 13].

One of the objectives of minimal-OS is to achieve an adjusted serum level of estradiol. Recently, studies shown high estradiol level, more than 3000 pg/ml in day of triggering final OOCYTE maturation of fresh ET cycles, could be predisposed to preeclampsia, intrauterine growth restriction, gestational hypertension, and small for gestational age infants [14].

In this study, the highest serum levels of estradiol in the day of triggering final OOCYTE maturation pertained to one of the two minimal-OS protocols. On the other hand, the lowest serum levels of estradiol were seen in the Mild-HMG and Minimal-HMG groups. Therefore, the administered gonadotropin type is an effective factor in the adjusting the serum estradiol level on the day of triggering final OOCYTE maturation, regardless of the COS protocol (mild-OS vs. Minimal-OS). In other words, both OS protocols with HMG could adjust the serum estradiol level on the day of triggering final OOCYTE maturation. Karimzadeh et al. also achieved a lower level of serum estradiol in minimal-OS cycles; however, the minimal-OS protocol was compared to the common protocol of OS with GnRH-agonist in the patients with a good fertility prognosis. Moreover, the administered gonadotropin was a combination of r-FSH and u-HMG [15]. Casano et al. also reported a lower estradiol level in the Mild-FSH cycle in comparison with an OS with GnRH-agonist in non-PCOS patients [16]. Andersen et al. compared HP-HMG and r-FSH in patients with a good fertility prognosis subjected to OS cycles with GnRH-agonist and showed a lower estradiol level after OS with r-FSH [17]. Bosch et al. compared these two gonadotropins in the patients with a good fertility prognosis undergoing OS cycles with a GnRH-antagonist and observed a higher estradiol level in those stimulated with HP-HMG [18]. Requena et al. compared urinary and recombinant gonadotropins in OOCYTE donation candidates in the OS cycle with GnRH-agonist and observed a lower estradiol level in the recombinant gonadotropin group [19]. Devroey et al. also reported a higher estradiol level in the HP-HMG group as compared to the r-FSH group in patients with good fertility prognosis in OS cycle with GnRH-antagonist [20]. Based on these studies, OS with u-HMG showed a higher serum estradiol level in the good prognosis patients, regardless of the stimulation protocol (GnRH-antagonist or agonist). Finding presents is inconsistent with the results of the present study, which can be attributed to the target group, i.e. patients with PCOS.

The present study showed that Minimal-FSH resulted the highest serum progesterone level on the day of triggering final OOCYTE maturation; whereas, the other three protocols were not significantly different in this regard. Given the effects of serum progesterone level on the fertility outcome on the day of triggering final OOCYTE maturation, Minimal-FSH is not an appropriate protocol. Andersen et al. showed that the progesterone level in OS GnRH-agonist cycles with r-FSH is higher than with the u-HMG [17]. Bosch et al. confirmed finding presents in OS cycles with GnRH-antagonist [18]. Devorey et al. and Requena et al. reported no significant difference in the serum progesterone levels between the OS cycles with u-HMG and r-FSH [19, 20].

This study showed that Mild-HMG requires the highest gonadotropin dose; whereas, Minimal-FSH requires a lower gonadotropin dose. It is interesting that despite the least dose of gonadotropin in the Minimal-FSH protocol in comparison with the other three protocols, it has the highest serum estradiol level. Therefore, gonadotropin dose < 150 IU may be more prudent. It is worth noting that the OS protocol does not affect the number of prescribed antagonists, that no difference was observed between the four study groups in this regard. The lower gonadotropin dose in mild-OS or minimal-OS has been reported as the advantage of these two protocols [15, 16, 21–25]; however, most studies reported a higher dose of u-HMG vs. r-FSH in mild-OS or minimal-OS [17–20, 26].

In a meta-analysis CC combined with gonadotropins and GnRH-antagonist versus conventional OS without CC, CC combined with GnRH-antagonist is likely to reduce the gonadotropin dosage [8], which is consistent with our results in the present study. In a meta-analysis of HP-HMG and r-FSH for OS in IVF cycles, who showed that the use of HP-HMG is associated with a higher prescribed dose of gonadotropin [10]; whereas, in our study mild-OS is a determinant factor of gonadotropin dose.

This study illustrated that Mild-FSH group possessed the shortest OS duration. Regardless of the type of prescribed gonadotropin, minimal-OS protocols were associated with a lower prescribed dose of gonadotropin without increasing the OS duration, which is an advantage in using these protocols. The meta-analysis of OS protocols, with and without CC, demonstrated a reduction in the OS duration in the presence of CC [8], which is inconsistent with the finding presents.

This study showed no significant difference in the endometrial thickness on the day of triggering final OOCYTE maturation among groups. Finding presents rejects the negative effects of CC on the endometrial thickness in the minimal-OS cycles. Karimzadeh et al. did not observe any difference in endometrial thickness between minimal-OS with CC and common OS with GnRH-agonist [15]. This is consistent with the finding of Devroey et al. study that compared different types of gonadotropins [20].

The present study showed that the Minimal-FSH is associated with the highest number of obtaining OOCYTE, OOCYTEs at the MII stage, number of 2PN, number of obtained embryos, and number of frozen embryos. The Mild-FSH was second to Minimal-FSH in these factors. It can be concluded that the type of prescribed gonadotropin (r-FSH) is the main factor in the obtaining a higher amount of OOCYTE and embryos in an OS cycle in the PCOS patients. It is worth noting that although the Mild-HMG and Minimal-HMG protocols were associated with a lower number of OOCYTE and embryo in comparison with the two other protocols, the number of obtaining OOCYTEs and embryos was in the acceptable range. Considering the dose of prescribed gonadotropin, the patients in Minimal-HMG protocol received less gonadotropin. Although, the Mild-HMG and Minimal-HMG were associated with a lower number of frozen embryos in comparison with the two other protocols, the patients can freeze their embryos. The difference in the obtaining embryos in our groups also applies to the embryos quality and has been associated with a significant difference in the number of high-quality embryos. In addition, Mild-HMG has been associated with the least number of high-quality embryos. However, this difference in good and poor quality is less tangible. Rinaldi et al. showed no significant difference between the mild-OS protocol with GnRH-antagonist and OS with GnRH-agonist in the number of embryos and their quality among patients with a history of OHSS [23]. Stimpfel et al. and Baart et al. also reported a higher number of OOCYTE in OS cycles with GnRH-agonist and a higher number of high-quality embryos in the mild-OS cycles with GnRH-antagonist in the patients with a good fertility prognosis [21, 24]. Devroey et al., Ziebe et al., Andersen et al., and Requena et al. also compared the prescribed gonadotropin and obtained similar results in terms of number of obtaining OOCYTE in the OS cycles with r-FSH and a higher number of high-quality embryos after OS with u-HMG [17, 19, 20, 26]. The meta-analytical comparison of HP-HMG with r-FSH for OS in the IVF cycles showed a lower number of OOCYTE after HP-HMG administration [10]. However, Cochran’s meta-analysis and review study suggested the lack of a significant difference in the number of obtaining OOCYTE and their quality in the OS protocols with GnRH-antagonist, with and without CC [8, 13].

Despite of qualitative and quantitative differences among our groups in terms of administering gonadotropin dose, OS duration, and the number of OOCYTE and embryos, there is no significant difference in the rates of fertilization, implantation, and miscarriage. Finding presents is confirmed by meta-analyses that compared mild and minimal protocols with common protocols of OS with GnRH-agonist or antagonist. Also these meta-analyses on the role of administering gonadotropin confirmed our findings [6, 8, 13].

It is worth noting that the cancellation of the OS cycle due to inadequate ovarian response was observed only in the OS protocol with u-HMG. Although, this difference is not statistically significant, this finding present the form of prescribed gonadotropin as an effective factor in the cancellation of the OS cycle, regardless of the type of protocol. Recent meta-analysis showed that although the mild-os protocol reduced the gonadotropin administration dose and OHSS incidence, it is associated with a reduced number of obtained OOCYTE and also a considerable increase in the cancellation of OS cycles due to POR [5].

The present study showed that Minimal-FSH exposes more than 90% of the patients at the risk of OHSS, so they subjected to GnRH-agonist triggering with triptorelin 0.2 mg s.c and freeze all embryos. Given that the risk of OHSS is a serious concern in the patients with PCOS, the Minimal-FSH protocol is not recommended for OS in these patients. The risk of OHSS is higher among the patients in Mild-FSH group and Minimal-FSH group. Therefore, the incidence of OHSS is affected by the prescribed gonadotropin type, regardless of the administered protocol. However, previous meta-analyses reported that the administration of CC in the OS cycles with GnRH-antagonist reduced the incidence rate of OHSS [8, 13]. It is worth noting that the majority of comparative studies between the mild-OS or minimal-OS protocols with GnRH-agonist protocols indicate a reduced incidence of OHSS in favor of mild-os or minimal-OS protocols [15, 16, 23, 24].

The present study showed that the Minimal-HMG protocol, followed by the Mild-HMG protocol, present a greater chance of having fresh ET; however, the Mild-FSH is the preferred protocol based on the clinical pregnancy rate. In addition, Minimal-HMG is comparable to it. It is worth noting that the available meta-analyses do not show a significant difference between protocols or the type of gonadotropin in the pregnancy rate, which can be attributed to the difference in their target population with the present study [6, 8, 13].

The present study showed that the PCOS phenotypes have not a significant role in the IVF cycle outcome of patients with PCOS, regardless of the OS protocol.

As was mentioned earlier, the lack of a similar study on the PCOS patients, as the target population, was a research limitation to the comparison of the present study with other similar studies to determine the best OS protocol. The major research limitation was because of economic constrain that inhibited blinding. Given that there are scant RCTs on IVF in patients with PCOS, further studies are recommended.

## Conclusions

Based on the findings of the present study, the minimal-OS with u-HMG may be one of optimal methods of control OS in the patients with PCOS in respect to serum levels of estradiol on the day of triggering final oocyte maturation, total dose of prescribed gonadotropin, the optimal number of oocytes and embryos obtained, rate of clinical pregnancy and the incidence of OHSS risk.

## Data Availability

All data generated or analyzed during this study are included in this published article.

## List of abbreviations

IVF: In vitro fertilization
PCOS: Polycystic ovarian syndrome
r-FSH: Recombinant follicle stimulating hormone
u-HMG: Urinary human menopausal gonadotropin
OHSS: Ovarian hyper stimulation syndrome
OI: Ovulation induction
ART: Assisted reproductive treatment
OS: Ovarian stimulation
GnRH: Gonadotropin-releasing hormone
Mild-OS: Mild ovarian stimulation
Minimal-OS: Minimal ovarian stimulation
LH: Luteinizing hormone
POR: Poor ovarian response
ISMAAR: International Society for Mild Approaches in Assisted Reproduction
CC: Clomiphene citrate
LBR: Live birth rate
CPR: Clinical pregnancy rate
HP-HMG: Highly purified HMG
RCTs: Randomized control trials
IUI: Intrauterine insemination
BMI: Body mass index
Minimal-FSH: Minimal-OS with r-FSH
Minimal-HMG: Minimal-OS with HP-HMG
Mild-FSH: Mild-OS with r-FSH
Mild-HMG: Mild-OS with HP-HMG
ET: Embryo transfer
RCOG: Royal College of Obstetricians & Gynaecologists
AMH: Anti mullerian hormone
Beta-HCG: Beta-human chorionic gonadotropin
MII: Metaphase 2
2PN: 2 pronuclear
ANOVA: Analysis of variance
CONSORT: Consolidated Standards of Reporting Trials
